# Role of tumor cell senescence in non-professional phagocytosis and cell-in-cell structure formation

**DOI:** 10.1186/s12860-020-00326-6

**Published:** 2020-11-07

**Authors:** Dorian Gottwald, Florian Putz, Nora Hohmann, Maike Büttner-Herold, Markus Hecht, Rainer Fietkau, Luitpold Distel

**Affiliations:** 1 Department of Radiation Oncology, Universitätsklinikum Erlangen, Friedrich-Alexander-Universität Erlangen-Nürnberg, D-91054 Erlangen, Germany; 2 Department of Nephropathology, Institute of Pathology, Universitätsklinikum Erlangen, Friedrich-Alexander-Universität Erlangen, Nürnberg, Germany

**Keywords:** Cell-in-cell, Non-professional phagocytosis, Senescence, Cannibalism, Entosis, Rectal cancer, Camptothecin, Prognostic factor and survival

## Abstract

**Background:**

Non-professional phagocytosis is usually triggered by stimuli such as necrotic cell death. In tumor therapy, the tumors often disappear slowly and only long time after the end of therapy. Here, tumor therapy inactivates the cells by inducing senescence. Therefore, study focused whether senescence is a stimulus for non-professional phagocytosis or whether senescent cells themselves phagocytize non-professionally.

**Results:**

Senescence was induced in cell lines by camptothecin and a phagocytosis assay was performed. In tissue of a cohort of 192 rectal cancer patients senescence and non-professional phagocytosis was studied by anti-histone H3K9me3 and anti-E-cadherin staining. Senescent fibroblasts and pancreas carcinoma cells phagocytize necrotic cells but are not phagocytized. In the tissue of rectal carcinoma, senescent cells can phagocytize and can be phagocytized. A high number of senescent cells and, at the same time, high numbers of non-professional phagocytizing cells in the rectal carcinoma tissue lead to an extremely unfavorable prognosis regarding overall survival.

**Conclusion:**

Senescent cells can be non-professionally phagocytized and at the same time they can non-professionally phagocytize in vivo. In vitro experiments indicate that it is unlikely that senescence is a strong trigger for non-professional phagocytosis. Combined high rates of non-professional phagocytosis and high rates of senescence are an extremely poor prognostic factor for overall survival.

## Background

Radiation therapy of cancer achieves its therapeutic effect through the induction of different types of cell death. The predominant types of cell death are apoptosis and necrosis, which are also the most widely discussed types of cell death recognized in current morphological nomenclature [1]. Apoptotic and necrotic cell death as well as most other kinds of cell death mostly lead to a rapid elimination of cell remnants. In striking contrast, however, it is well known from radiotherapy that most malignancies do not resolve during multi-week fractionated radiation treatment. Indeed, most malignancies require several weeks after the end of treatment to show volumetric tumor regression in clinical and imaging assessment [2, 3]. In rectal cancer for example, Habr-Gama et al. found a median time interval of 18.7 weeks from completion of radiotherapy to complete endoscopic clinical response [4]. An alternative route to tumor inactivation is instead of initiating cell death, to induce non-proliferative cells by triggering so-called premature senescence [5]. It means that cancer cells initiate a programmed sequence of events leading to a phenotype of a stable and long-term loss of proliferative capacity, despite continued viability and metabolic activity [6]. Changes observed in senescent cells include the activation of the tumor suppressor network, morphological changes, altered chromatin structure and an altered spectrum of secreted factors [7, 8]. Cancer therapy and especially radiochemotherapy usually causes senescence [9–11].

Senescent cells thus could be the reason for the delayed removal of cancer cells after RT. Senescent cells cause a pro-inflammatory response known as senescence-associated secretory phenotype (SASP) [12, 13]. This inflammation may clear the senescent cells by immune-mediated phagocytosis [14, 15]. Another option to clear cells is the phagocytosis by non-professional phagocytes. It was shown that various normal tissue cells [16–19] and cancer cells [20–25] have the capability to engulf apoptotic, autophagic or necrotic cells. This leads to a cell-in-cell (CIC) phenotype and a subsequent digestion of the phagocytized cells. In cancers the frequently observed phenomenon of cell-in-cell structures by non-professional phagocytosis has also been referred to as “cell cannibalism” and describes the uptake and elimination of malignant cells by other cancer cells. Cell cannibalism is a very frequent finding in cancer [22, 26]. A so far not resolved question is whether senescent cells are cleared by non-professional phagocytes and if the increase of cell-in-cell structures and senescence in pathologic specimens are somehow related.

The aim was to study the involvement of senescence in cell-in-cell, either as a recipient cell or as an engulfing cell. We used a pancreatic carcinoma cell line and two primary fibroblast cell lines to act as non-professional phagocytes. It was studied whether senescent cells are preferred targets of non-professional phagocytosis or entotic cell death. In addition, using histologic samples from rectal cancer patients obtained prior to and after radiochemotherapy, the prevalence and prognostic significance of non-professional phagocytosis in rectal cancer as well as the association between tumor cell senescence and cell-in-cell structure formation were studied.

## Results

### Non-professional phagocytosis

**Fig. 1:**
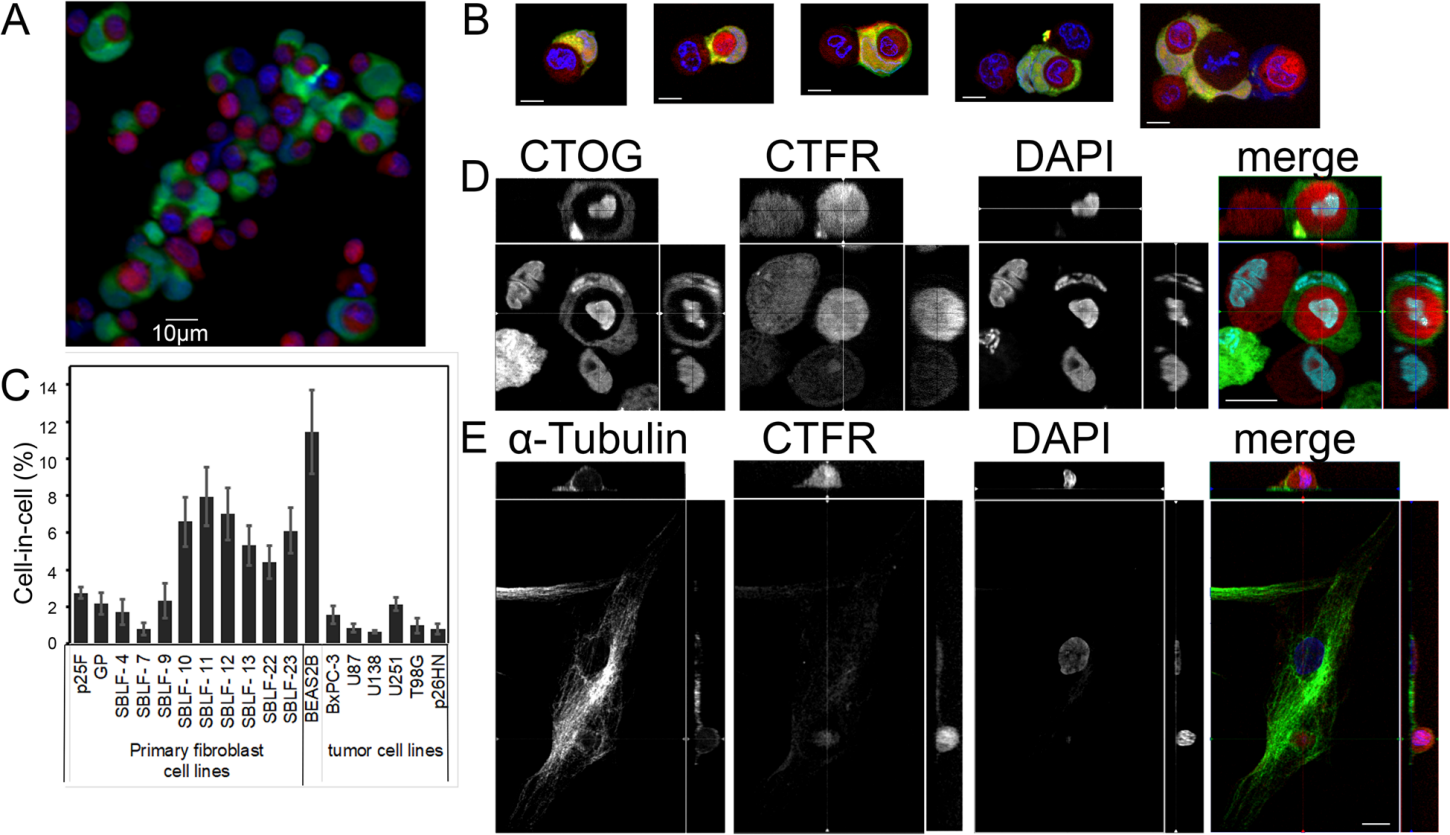
Heat-treated cells (56 °C, 45 min) were engulfed by non-professional phagocytes. Heat-treated cells stained red with CTFR and living cells stained green with CTOG. Nuclei stained blue with DAPI. **a** Normal epithelial lung tissue cells BEAS2B. **b** Different phases of cell attachment during phagocytosis in BEAS2B lung epithelial cells. **c** Cell-in-cell rates in different primary fibroblasts and tumor cell lines. Z-stack images were acquired by optical sectioning of 60 images. Image planes of the X, Y and Z plane are included. **d** BEAS2B epithelial lung cells and **e** SBLF-4 primary fibroblast. Instead of the CTOG staining the α-tubulin staining was used. Scale bars: 10 μm

Dead cells induced by heat were non-professionally phagocytized by living epithelial lung cells (BEAS2B). Cell death was induced by overheating to 56 °C for 45 min (Fig. 1a). The living cells adhered to the dead cells and phagocytized them within two to 10 h (Fig. 1b). Phagocytotic rates ranged from less than 0.65 to 11.5%. Normal tissue cell lines (mean value 5.6%) phagocytized more than tumor cell lines (1.2%) (*p* = 0.001) (Fig. 1c). In the process of phagocytosis, the overheated cell was completely engulfed by the living cell (Fig. 1d, e).

### Senescence in tumor and normal-tissue cell lines

**Fig. 2:**
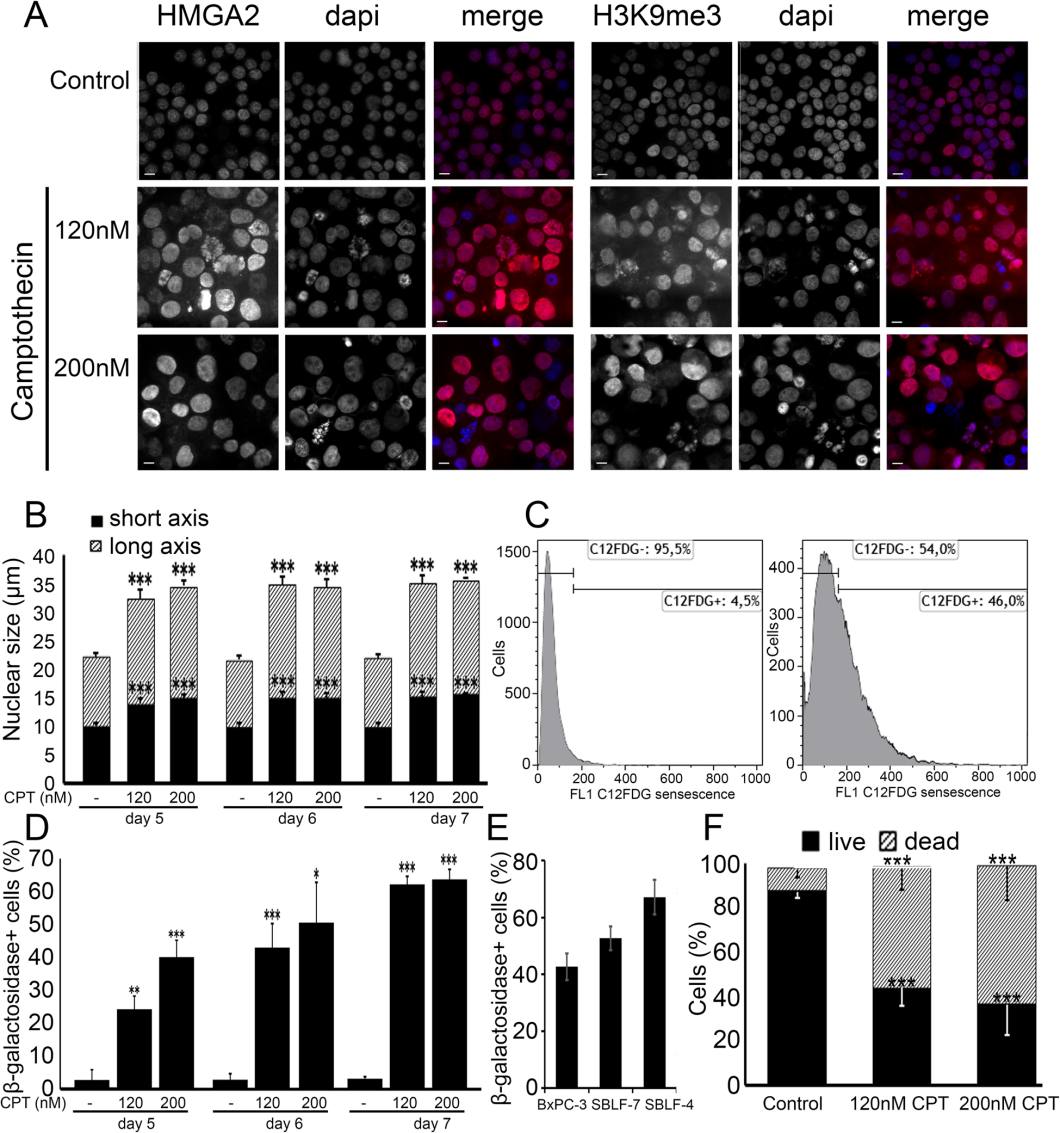
**a** Immunofluorescence staining of the senescence markers HMGA2 and H3K9me3 in red without and after 6 days treatment with camptothecin (CPT) 120 nM and 200 nM for senescence induction in pancreas carcinoma cells BxPC-3. The cell nuclei were stained with DAPI (blue). Scale bar 10 μm. **b** Cell nucleus length and width of BxPC-3 cells after 6 days CPT treatment. **c** C_12_FDG β-galactosidase activity in untreated and 120 nM treated BxPC-3 cells on day 6. **d** Activity of the acidic β-galactosidase in pancreas carcinoma cells after CPT treatment on days 5, 6 and 7 using flow cytometry. The graphs represent the mean values from three independent experiments ± standard deviation, *p*-values: * < 0.05, ** < 0.01, *** < 0.001. **e** C_12_FDG β-galactosidase activity in 120 nM treated BxPC-3, SBLF-7 and SBLF-4 cells on day 6. **f** Flow cytometric determination of the amount of Annexin V−/7AAD- (living cells) compared to all others (dead cells) 7 days after 120 nM camptothecin treatment. p-values were determined from a two-tailed unpaired Mann Whitney U test: ** < 0.01, *** < 0.001

The aim was to study whether senescence is a trigger for non-professional phagocytosis observed for heat-induced cell death and whether senescent cells are being phagocytized by non-senescent cells. The chemotherapeutic agent camptothecin (CPT) was used to trigger senescence in BxPC-3 cells. After 6 days of incubation with 120 nM or 200 nM CPT cells were assessed for senescence induction by detection of high-mobility group AT-hook 2 (HMGA2) protein and Histone 3 tri-methylated at the 9th lysine residue (H3K9me3) heterochromatic foci formation, flow cytometric β-galactosidase staining as well as the estimation of the nuclear size in both axes. A clear change of the staining pattern (Fig. 2a) and a distinct increase in nuclear size in both axes (Fig. 2b) was detected.

Additionally, the β-galactosidase activity was analyzed and a distinct increase up to day six by 120 nM CPT was found (*p* < 0.05) (Fig. 2c, d). Thus, the 120 nM CPT on day 6 induced a β-galactosidase+ activity in BxPC3+ cells of 42.7 (standard deviation ±4.7%), in SBLF-7 of 52.7 ± 4.2% and in SBLF-4 of 67.1 ± 6.0% (Fig. 2e). Cells with a high percentage of senescent cells on day 7 also had increased levels of Annexin V and 7-Aminoactinomycin (7AAD) positivity (Fig. 2f). We selected the BxPC-3 cell line and the fibroblasts cell cultures SBL-F7 and SBLF-4 having 120 nM CPT induced senescence rates of 78.0% (standard deviation ±4.2%), 74.1% ± 7.2 and 97.8% ± 8.8, respectively (Additional file 1: Figure 1).

**Fig. 3:**
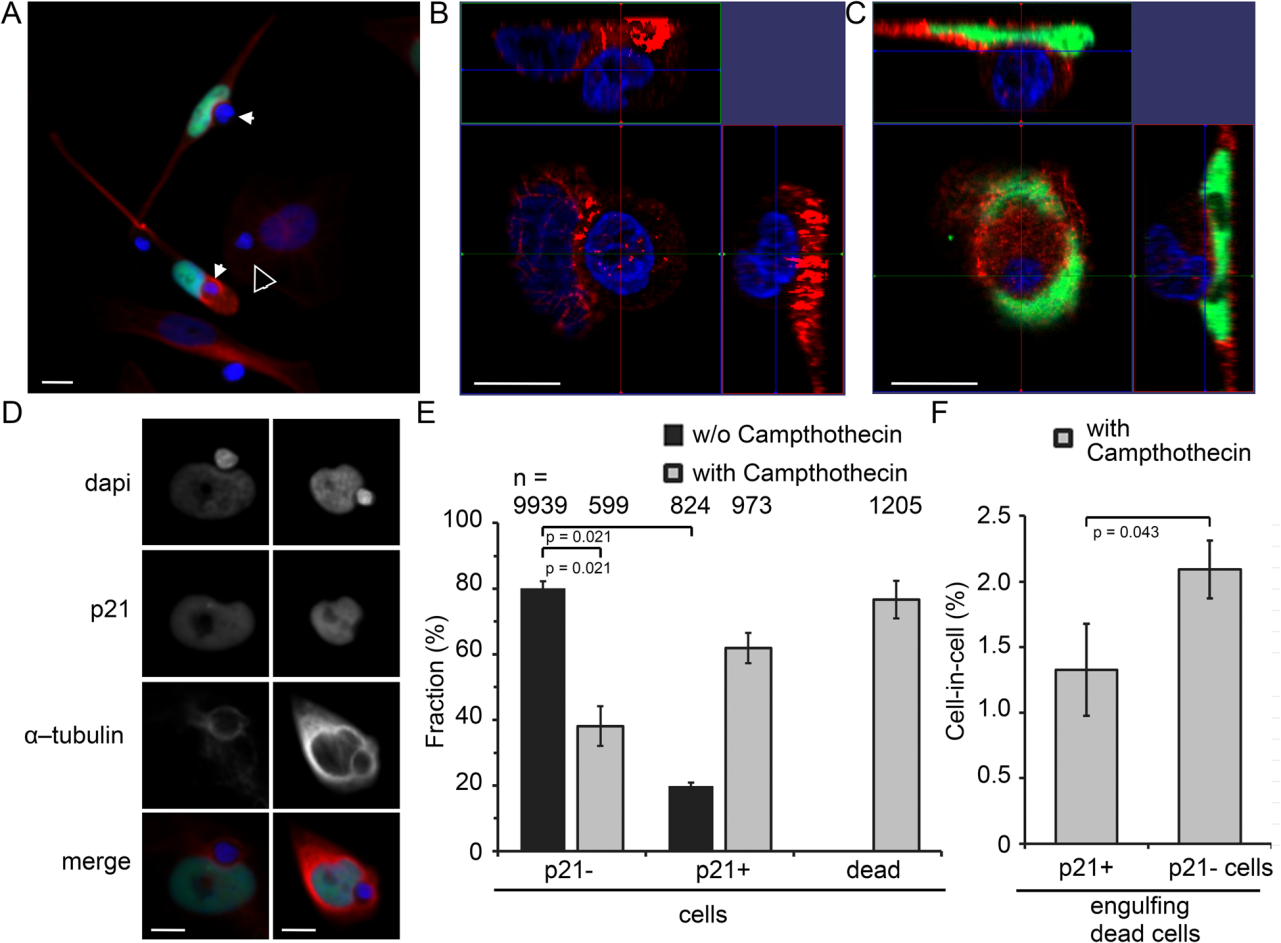
**a** Immunofluorescence staining after 6 days treatment of BxPC-3 cells with 120 nM campthotecin. p21 (green) was used as senescence marker, α-tubulin (red) as cytoskeleton marker and dapi (blue) for nuclear staining. Small blue cells are dead cells that were hyperthermized for 45 min at 56 °C. Filled arrowheads represent CIC of dead cells during the process or after engulfing. Open arrowheads represent dead cells engulfed by living, non-senescent cells. Orthogonal sections of one (**b**) non-senescent and one (**c**) senescent cell with an engulfed dead cell. Image planes of the X, Y and Z planes are visualized. **d** p21+ cells with an enclosed dead cell. **e** The proportion of non-senescent living blue cells (p21-) and senescent living p21+ green cells was analyzed. The dead cells were presented as a percentage of the living cells. **f** Cells treated with campthotecin and the percentage of dead cells included in p21+ senescent cells and in p21- non-senescent cells. *n* = number of cells counted in at least three independent experiments. Differences were analyzed by a two-tailed unpaired Mann-Whitney U. Scale bars: 10 μm

Alternatively, the induction of senescence in BxPC-3 cells was studied using immunostaining (Fig. 3a-d). After treatment with 120 nM campthothecin, the percentage of p21 staining as senescence marker increased from 19.9 to 80.1% (*p* = 0.021). With 76.6% there were slightly less dead cells present compared to living cells (Fig. 3e) (*p* = 0.014). In the BxPC-3 cell line, the CIC rates of non-senescent cells and senescent cells were determined. 1.3% of p21+ senescent cells and 2.1% of non-senescent cells had at least one dead cell phagocyted (*p* = 0.043) (Fig. 3f).

### Association of cell-in-cell structure formation and senescence in tumor and normal-tissue cell lines

**Fig. 4:**
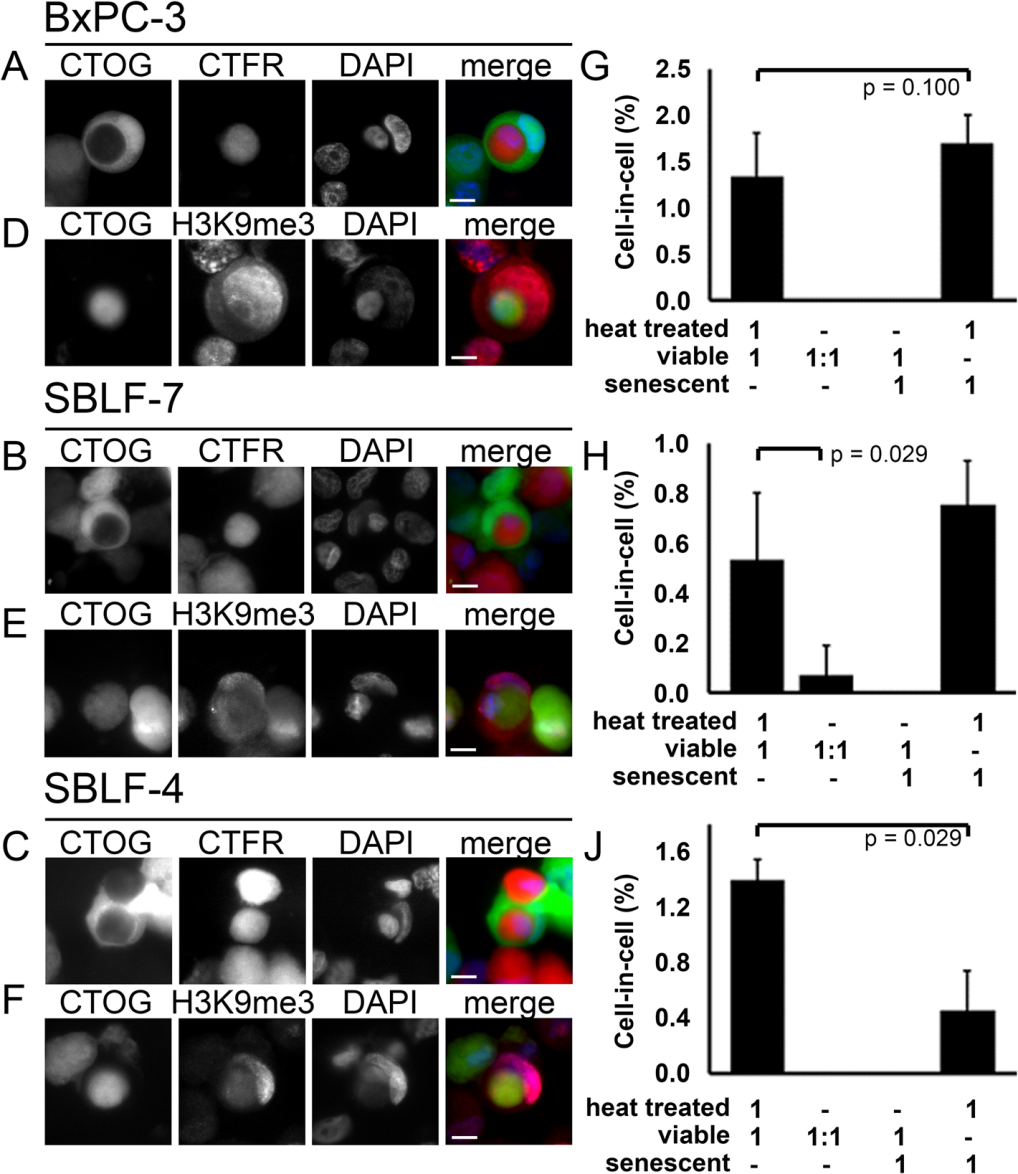
Representative microscopic images of typical cell-in-cell structures in phagocytosis experiments with (**a**, **d**) pancreas carcinoma cell line (BxPC-3) and fibroblast cell lines (**b**, **e**) (SBLF-7) and (**c**, **f**) (SBLF-4). Viable CTOG-stained (green) cell, which completely encloses a hyperthermia-damaged, CTFR-stained (red) cell (**a**-**c**). Senescent H3K9me3-stained (red) cells that completely enclose a hyperthermia-damaged CTOG-stained (green) cell (**d**-**f**). Cell nuclei were stained with DAPI (blue). Scale bars 10 μm (40x objective). In bar charts (**g**), (**h**) and (**j**) the cell-in-cell rates occurring in the respective cell lines on the left were shown for different combinations of senescent, living and heat-treated dead cells as indicated

Immunostaining was used to study non-professional phagocytosis of the three cell lines. Either cells were stained green or red by fluorescent live-cell stains (Fig. 4a-c). H3K9me3 antibodies were used to identify senescence cells (Fig. 4d-f). Only H3K9me3-positive cells were counted, which have had phagocytized CTOG-positive cells. No CIC events were observed, suggesting that cells showing signs of premature senescence are not phagocytized in vitro by viable cells. Conversely, it was not observed that living cells or senescent cells phagocytized living cells. H3K9me3+ senescent cells were co-incubated with CTOG+ necrotic cells and a similar high frequency of phagocytic event as for the viable cells (0.95%) was found (Fig. 4g-j). It indicates that senescent cells are capable of phagocytosis at comparable frequencies to non-senescent cells.

### Cell-in-cell structures in clinical rectal cancer tissue samples

**Fig. 5:**
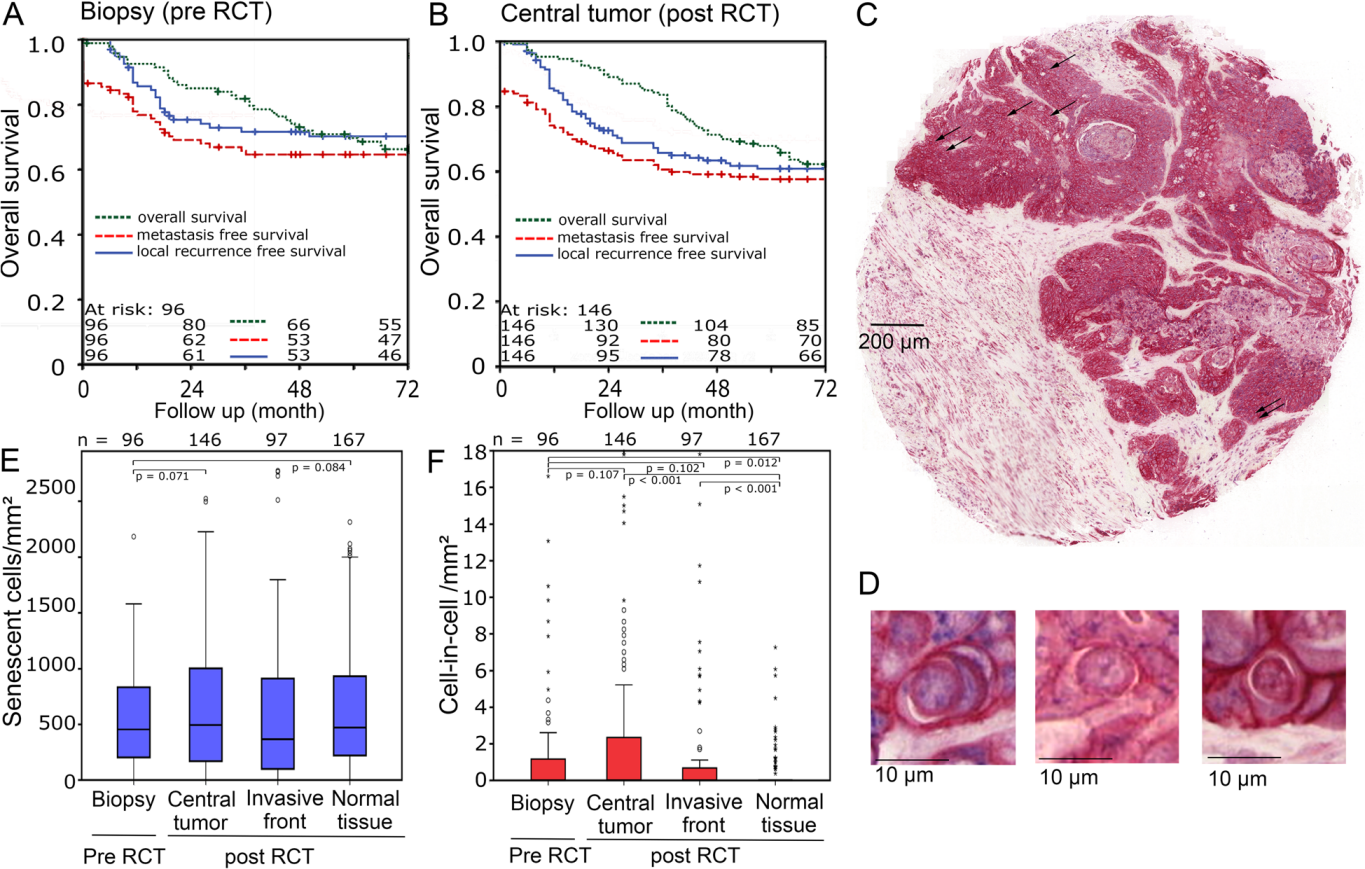
Kaplan Meier plots for overall survival, metastasis free survival and local recurrence-free survival for the cohort of patients (**a**) of which tissue micro arrays from pretherapeutic biopsies and (**b**) of post-RCT tumor resection were available. **c** Example of a micro array spot of rectal carcinoma tissue with a diameter of 2 mm that was immunohistochemically double stained by nuclear anti-H3K9me3 (blue, senescent) and anti-E-Cadherin (red membranous). Arrows indicating cell-in-cell events. **d** Individual cell-in-cell events. **e** Frequency of counted intraepithelial senescent cells/mm^2^ of patients in pre-RCT biopsy and post-RCT central tumor, invasive tumor front and normal tissue. **f** Frequency of counted intraepithelial cell-in-cell phenomena/mm^2^ in patients in pre-RCT biopsy and post-RCT central tumor, invasive tumor front and normal tissue area. Differences between groups were analyzed by unpaired Student’s t-test

The frequency and prognostic relevance of cell-in-cell structures and senescent cells was studied in a rectal cancer cohort that was treated with neoadjuvant radiochemotherapy (RCT). A total of 96 patients with Tissue Micro Array (TMA) samples of tumor biopsy (“biopsy”), obtained during pretreatment endoscopy, were analyzed. In addition, TMA samples from the resected tumor at 6 weeks post-RCT were used for analysis. TMAs from these surgical specimens were available in 146 patients from the tumor core “central tumor”, in 97 patients in the tumor invasion zone (“invasive front”) and in 167 patients in surrounding normal tissue (“normal tissue”). The clinical and histological characteristics of the cohort are given in Table 1. Patients having pre-RCT biopsies, had a 5-year overall survival of 69.8%, a metastasis-free survival of 64.5% and a local recurrence-free survival of 70.1%, respectively (Fig. 5a). Post-RCT patients had a 5-year overall survival of 67.8%, a metastasis-free survival of 57.8% and a local recurrence-free survival of 61.1%, respectively (Fig. 5b). All TMAs were stained by antibodies, for anti-H3K9me3 (blue, nuclear) to detect senescent cells and for anti-E-Cadherin (red, membranous) to detect cell-in-cell structures (Fig. 5c, d).

**Table 1:** Clinical characteristics of rectal cancer patients

		Pre RCT biopsies	Post RCT tumor resections
		n (%)	n (%)
Variable	All patients	96 (100)	146 (100)
Gender	male	63 (65.6)	110 (75.3)
	female	33 (34.4)	36 (24.7)
Age at diagnosis	mean	64 y (35–93 y)	63 y (33–84 y)
UICC stage	I	7 (7.3)	8 (5.5)
	II	24 (25)	34 (23.3)
	III	52 (54.2)	81 (55.5)
	IV	13 (13.5)	23 (15.8)
Grading	G1	4 (4.2)	3 (2.1)
	G2	77 (80.2)	116 (79.5)
	G3	15 (15.6)	27 (18.5)
T stage	T1	4 (4,2)	3 (2.1)
	T2	13 (13.5)	18 (12.3)
	T3	72 (75)	105 (71.9)
	T4	7 (7.6)	20 (13.7)
N stage	N0	32 (33.3)	48 (32.9)
	N1	64 (66.7)	98 (67.1)
	N2	0 (0)	0 (0)
	N3	0 (0)	0 (0)
M stage	M0	83 (86.5)	123 (84.2)
	M1	13 (13.5)	23 (15.8)
Chemotherapy	5-FU mono	38 (39.6)	53 (36.3)
	Oxaliplatin - 5-FU	42 (43.8)	76 (52.1)
	other 5-FU-combinations	7 (7.3)	5 (3.4)
	others	9 (9.4)	12 (8.2)
Dworak regression grade	0	1 (1)	3 (2.1)
	1	8 (8.3)	9 (6.2)
	2	22 (22.9)	55 (37.7)
	3	53 (55.2)	79 (54.1)
	4	12 (12.5)	0 (0)

### Prevalence of senescence and cell-in-cell structures in different tumor compartments

The median number of epithelial senescent cells/mm^2^ was highest in the central tumor area (560 cells/mm^2^) followed by normal tissue (494 cells/mm^2^). In the biopsies and invasive front numbers of senescent cells were similar (360 cells/mm^2^ vs. 369 cells/mm^2^), respectively (Fig. 5e). The mean numbers of epithelial cell-in-cell structures/mm^2^ were most frequent in the invasive front (1.0 cell-in-cell/mm^2^) followed by the central tumor region (0.72 cell-in-cell/mm^2^) and biopsies (0.56 cell-in-cell/mm^2^). In normal tissue cell-in-cell phenomena were least common (0.08 cell-in-cell/mm^2^) (Fig. 5f).

### Prognostic significance of cell-in-cell and senescence rates in clinical rectal carcinoma tissue samples

**Fig. 6:**
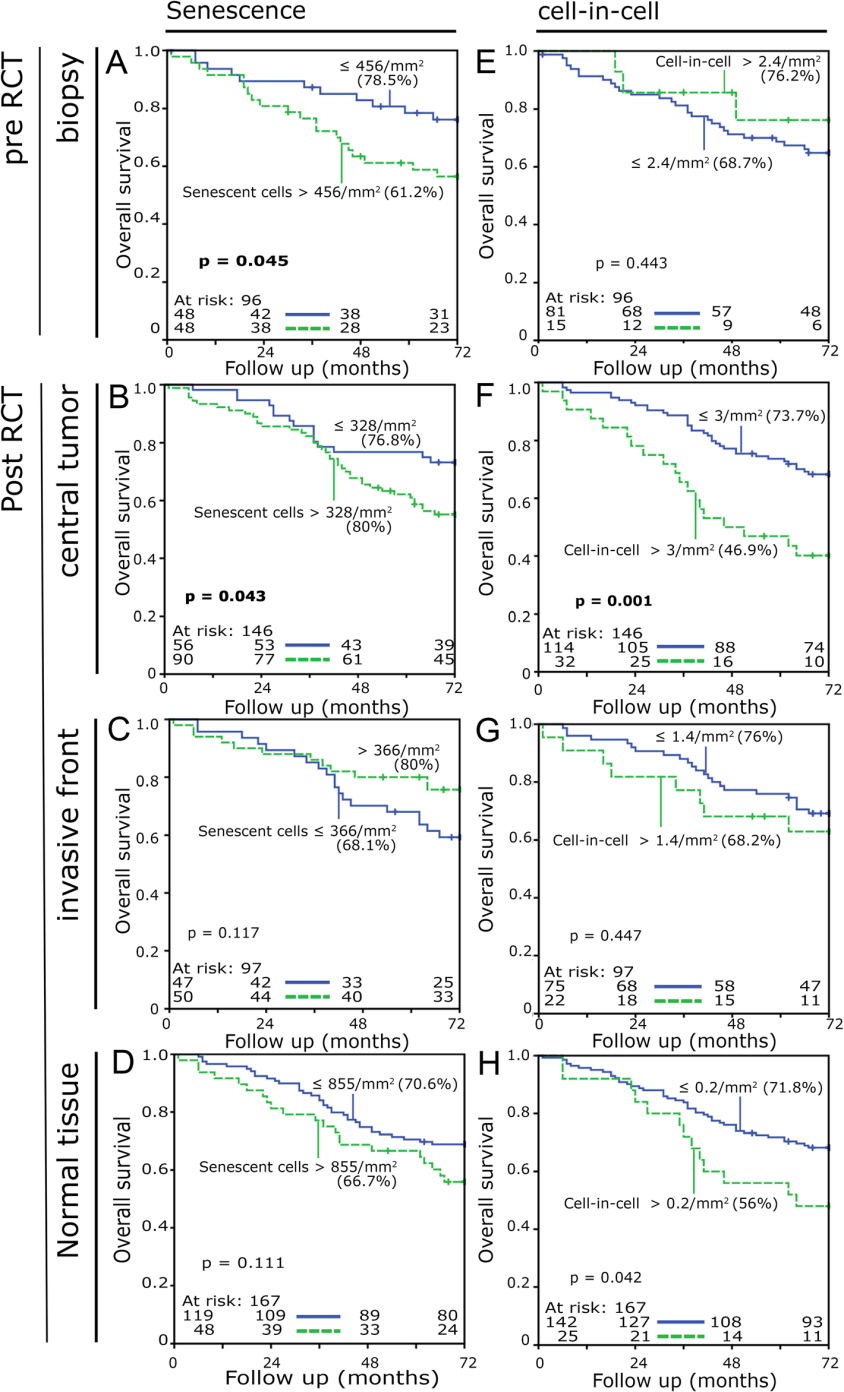
Prognostic significance of intraepithelial senescent cell density/mm^2^ and cell-in-cell phenomena density/mm^2^ in Kaplan Meier plots for overall survival. The cut-off values were determined by the ROC curve analysis. This resulted in specific cut off values for each individual analysis. Five-year survival rates are given in brackets after the designation of the corresponding cut-off values. **a** Senescent cell density in tissue micro arrays of biopsies, **b** central tumor (**c**) invasive tumor front and (**d**) normal tissue from tumor resection. **e** Cell-in-cell density in tissue micro arrays of biopsies, **f** central tumor (**g**) invasive tumor front and (**h**) normal tissue from tumor resection. Survival analysis was performed using the Kaplan-Meier method with log-rank test

The prognostic relevance of senescent cells and cell-in-cell structures for the following outcomes was analyzed: overall survival, metastasis-free survival, local recurrence-free survival and tumor-specific survival. For each outcome the cut-off values were determined by receiver operating characteristic (ROC) analysis resulting in specific cut off values for each individual analysis. Overall survival was clearly favorable for patients with a low number of senescent cells in pretreatment biopsies (5-year overall survival, 78.5% v. 61.2%, *p* = 0.045) (Fig. 6a) as well as central tumor areas (80% v. 76.8%, *p* = 0.043) (Fig. 6b).

Senescent cells in the invasive tumor front or normal tissue had no prognostic relevance, however (Fig. 6c, d). Cell-in-cell in biopsies were not significantly associated with prognosis. Patients having less than three cell-in-cell structures/mm^2^ in the central tumor had a clearly improved overall survival (73.3% v. 46.9%, *p* = 0.001) (Fig. 6e, f, g). Patients having less than 0.2 cell-in-cell structure/mm^2^ in their normal tissue (Fig. 6h) also had a distinctly improved prognosis (71.8% v. 56.0%, *p* = 0.042).

Moreover, low numbers of senescent cells in the biopsies (86.3% v. 74.4%, *p* = 0.046) and low numbers of cell-in-cell structures (80.4% v. 65.7%, *p* = 0.015) and senescent cells (89.0% v. 70.6%, *p* = 0.014) in the central tumor area were associated with improved tumor-specific survival (Additional file 2: Figure 2). High numbers of senescent cells in the invasive tumor front were a predictor of a favorable local recurrence-free survival (73.3% v. 49.3%, *p* = 0.024, Additional file 3: Figure 3). Patients with high senescent cell rates in biopsies (53.7% v. 75.6%, *p* = 0.021) and central tumor (50.3% v. 73.7%, *p* = 0.013) developed metastatic disease significantly more frequently than patients with low rates (Additional file 4: Figure 4).

### Association of senescence with cell-in-cell structures in rectal cancer tissue samples

**Fig. 7:**
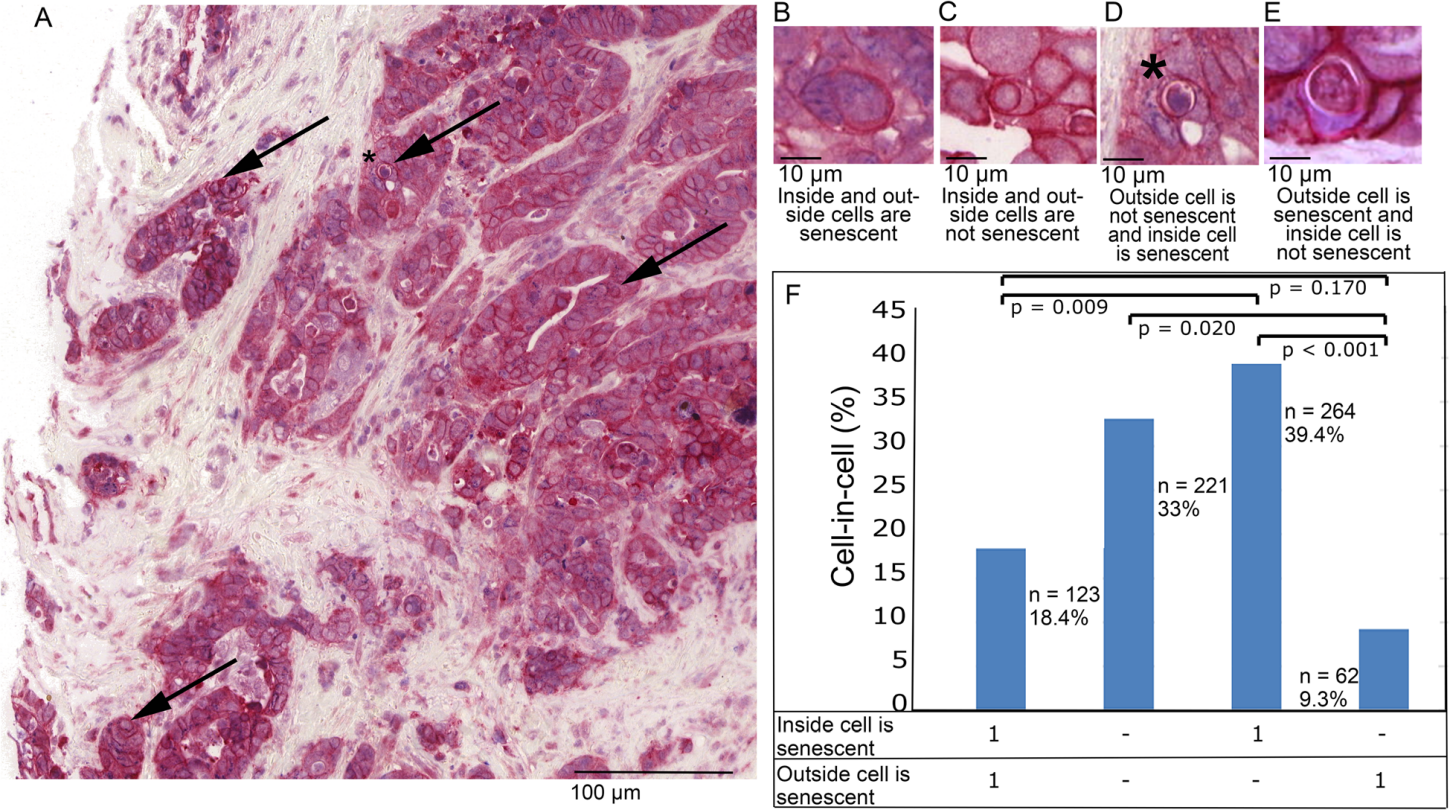
**a** Part of a TMA immunohistochemical double stained for senescence by nuclear anti-H3K9me3 (blue) and membranous anti-E-Cadherin (red) in rectal cancer. Arrows indicating cell-in-cell events. Zoomed images of the tissue micro array, **b** a cell-in-cell event in which both involved cells are senescent, **c** a cell-in-cell event in which both involved cells are not senescent, **d** engulfed cell is senescent (asterisk marks the cell in the tissue micro array) and (**e**) a cell-in-cell event in which only the engulfing cell is senescent. **f** Distribution of senescence in the engulfing and in the engulfed cell in all observed cell-in-cell events. Differences were analyzed by the Student’s t-test

Each observed cell-in-cell structure (*n* = 670) was photographed and was analyzed according to whether the engulfed and/or phagocytotic cell was senescent (Fig. 7a-e).

Senescent cells were observed in 67% of all cell-in-cell structures. Most frequently senescent cells were engulfed by a non-senescent cell (39.4% of all cell-in-cell structures, Fig. 7b, f), followed by both participating cells not being senescent (33%, Fig. 7c, f) and both cells being senescent (18.4%, Fig. 7d, f). Non-senescent cells only rarely were engulfed by senescent host cells (9.3%, Fig. 7e, f). This association was highly significant: Senescent cells were significantly more frequently internalized in cell-in-cell structures than constituting the external host cell (57.8% vs. 27.6%, *p* < 0.001 Fisher’s exact test).

### Combination of cell-in-cell and senescence rates as prognostic factors

**Fig. 8:**
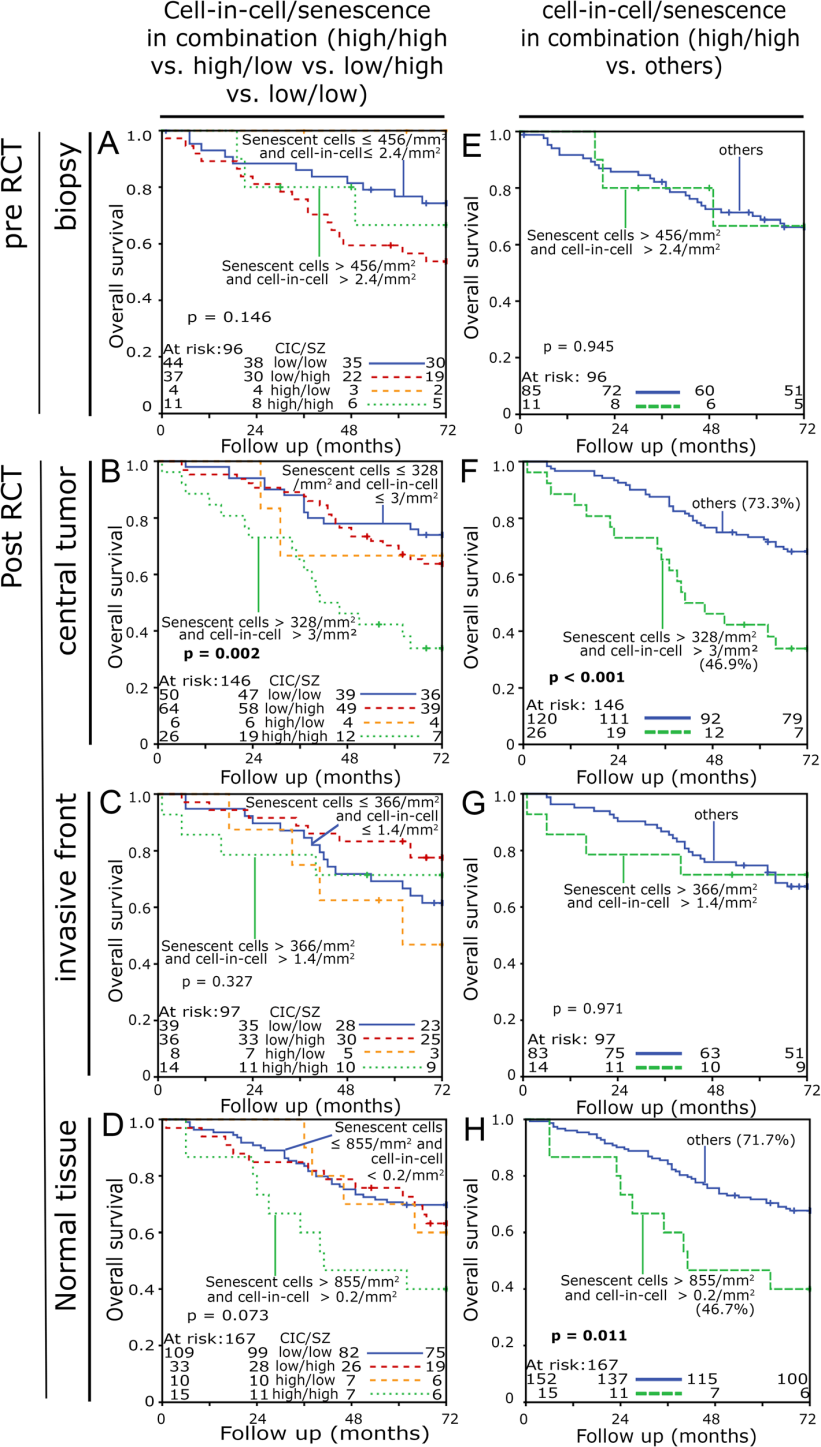
Kaplan Meier plots for overall survival of intraepithelial senescent cell density/mm^2^ and cell-in-cell phenomena density/mm^2^. The cut-off values were determined by the ROC curve analysis. This resulted in specific cut off values for each individual analysis. The five-year survival rate is given in brackets after the designation of the corresponding cut-off values. All graphs in the left column show high and/or low rates of senescent cells combined with high and/or low cell-in-cell events. **a** biopsies. **b** central tumor, **c** invasive tumor front and **d** normal tissue from the tumor resection. The graphs in the right column compare high senescence and high cell-in-cell rates to all other combinations. **e** biopsies. **f** central tumor, **g** invasive front and **h** the normal tissue from the tumor resection. Five-year survival rate is shown in brackets (**f**) and (**h**). Statistical significance was checked by the log-rank test

In addition, the prognostic relevance of combined rates of senescence and cell-in-cell phenomena was studied. The combined group having high numbers of senescent cells and high cell-in-cell/mm^2^ rates was associated with a poor overall survival (Fig. 8a-d).

Therefore, high cell-in-cell and high senescent cell rates were compared to all others (Fig. 8e-f). Patients with high senescent and cell-in-cell rates in the central tumor had a particularly unfavorable prognosis (73.3% v. 46.9%) (*p* < 0.001, Fig. 8f). Similarly, in the normal tissue patients with “high-high” rates showed an unfavorable prognosis (71.7% v. 46.7%) (*p* = 0.011) (Fig. 8h). Univariate and multivariate Cox’s regression analyses for overall survival were performed including clinical prognosticators and TNM stage. M stage (*p* < 0.001) and combined high cell-in-cell/mm^2^ with high senescence/mm^2^ in the tumor post RCT (*p* = 0.001) were independent prognostic factors for overall survival in multivariate analysis (Table 2).

**Table 2:** Univariate and multivariate analysis of overall survival according to Cox’s proportional hazards model

Rectal cancer	Univariate analysis			Multivariate analysis		
Variable	Hazard ratio	95% C.I.	*p*	Hazard ratio	95% C.I.	*p*
T stage (T4 [*n* = 21] v. T1/T2/T3 [*n* = 121])	0.596	0.272–1.306	0.196	0.555	0.254–1.213	0.140
N stage (N+ [*n* = 101] v. N0 [*n* = 41])	0.732	0.4–1.337	0.309	0.777	0.433–1.395	0.398
M stage (M+ [*n* = 23] v. M0 [*n* = 119])	5.233	2.682–10.208	**< 0.001**	4.495	2.469–8.183	**< 0.001**
Grading (3 + 4 [*n* = 27] v. 1 + 2 [*n* = 115])	1.329	0.674–2.619	0.411	–	–	–
Gender (female [*n* = 34] v. male [*n* = 108])	0.796	0.416–1.522	0.491	–	–	–
Age (> 58 years [*n* = 72] v. < 58 years [*n* = 70])	1.772	1.002–3.132	**0.049**	1.635	0.942–2.839	0.081
Cell-in-cell > 3/mm^2^ [*n* = 31] v. ≤ 3/mm^2^ [*n* = 111]	1.237	0.617–2.477	0.549	–	–	–
Senescence > 328/mm^2^ [*n* = 89] v. ≤ 328/mm^2^ [*n* = 53]	0.895	0.116–6.928	0.915	–	–	–
Cell-in-cell > 3/mm^2^ and senescence > 328/mm^2^ [*n* = 26] v. others [*n* = 116]	2.849	0.332–24.434	0.340	2.623	1.46–4.713	**0.001**

## Discussion

Cell lines with induced senescence were not phagocytized in vitro, but were able to phagocytise other cells very effectively. In contrast, naturally occurring senescent cells in rectal cancer tissue samples were phagocytized. Only 27.6% of phagocytic cells were senescent, whereas 57.8% of phagocytized cells presented a senescent state. Low cell-in-cell rates were associated with favorable overall survival. A high number of senescent cells and at the same time a high number of cell-in-cell structures in the tissue lead to an extremely unfavorable prognosis regarding overall survival.

The question whether senescent cells are a stimulus for phagocytosis has been answered contradictory. However, the results of the cellular in vitro investigations were unmistakable. In all three studied cell lines absolutely no senescent cells had been phagocytized. Since clear experimental conditions are given and a high percentage of senescent cells were available and no phagocytosis occurred at all, this strongly suggests that senescence is not a trigger for phagocytosis. In tissue, the properties of the cells are not so definite. Even if the cells have a senescent phenotype, they may have suffered a necrotic death afterwards. In particular, radiochemotherapy involves daily radiation for about 6 weeks and additional chemotherapy. In this way cells can be driven into senescence [27] and then these cells can be damaged even further so that they die as a consequence [28]. Death of senescent cells is mainly necrotic or other non programmed death [29, 30]. Therefore, the phagocytized cells could also be necrotically dead senescent cells. Since necrosis is a strong trigger for phagocytosis [19, 31], it can lead to subsequent engulfment of the senescent cells that have died necrotically. Necrotic cells cannot actively penetrate living cells but are actively ingested by living cells. An alternative explanation could be entosis, in which one cell actively penetrates another cell and causes its own uptake in the recipient cell [32]. It is thinkable that the observed cell-in-cell structures were formed by entosis and that this mechanism is applied preferentially by senescent cells. However, the experimental setup did not allow to assess this.

Another interesting issue is that heat-induced necrosis is a very strong trigger of non-professional phagocytosis. Sixty percent of cells treated with camptothecin were identified as dead in the Annexin V/7AAD assay. Astonishingly, however, in contrast to the cells killed at 56 °C, they were not phagocytized. This indicates that a difference exists that inhibits cells killed by camptothecin to be non-professionally phagocytized and thus prevents cell-in-cell phenomena. Additionally, this observation indicates that senescence per se is not a distinct trigger of non-professional phagocytosis.

In general cell-in-cell structures are found both in normal tissue [31] and in tumor tissue [33]. A high rate of cell-in-cell structures in tumor tissue is a negative prognostic factor for example in breast carcinoma [20, 34], metastasis of melanoma [24], cytology of bladder cancer [35], head and neck cancer [25, 36], rectal cancer [25], oral squamous cell carcinoma [37] and lung adenocarcinoma [38]. In contrast, an increased incidence of homotypic cell cannibalism in ductal adenocarcinoma of the pancreas was associated with decreased metastatic potential and consequently was a positive prognostic factor [21]. Additionally, adherent wild-type cells can act efficiently as entotic hosts, suggesting that normal epithelia may engulf and kill aberrantly dividing neighboring cells [39]. High cell-in-cell rates and high senescence rates are prognostically unfavorable markers for rectal cancer patients in this study. Senescence occurred more frequently after RCT in the central tumor tissue. Cell-in-cell structures were also found more frequently in the central tumor. This study shows that high cell-in-cell rates in rectal carcinomas can be considered as an adverse prognostic marker for survival. A reason might be that the phagocytosis leads to an increased supply of substances to the tumor cells [40–42]. In addition, selection of particularly malignant tumor cells is likely to occur by competition, in which the ability to engulf neighboring cells could constitute a distinct advantage [43]. The poorer survival for patients with high senescence values could be explained by the senescence-associated secretory phenotype [44–47]. There could be an association between cell-in-cell structures and senescence. Hints for this are that senescent cell lines like fibroblasts and pancreas carcinoma cells phagocytize in vitro and that in vivo in 67% of cell-in-cell structures in rectal cancer TMAs senescent cells are involved. In the tissue of rectal cancer mainly senescent cells are phagocytized, but they can also phagocytize. The extremely poor survival of the subgroup with combined high senescence rates and high cell-in-cell rates is impressive. It was an independent marker for dramatically worse overall survival in rectal cancer patients. Prognostic markers are important for accurately individualizing therapies for rectal cancer patients, thereby reducing long-term costs and patient suffering [48, 49]. Given the strong prognostic significance in this investigation for rectal cancer patients, additional studies on cell-in-cell structures and senescence are clearly warranted. We would also have liked to study non-professional phagocytosis in normal tissue. However, in these tissues the CIC rates are extremely low [31] and accordingly the events with senescence are much lower. In the tumor surrounding normal tissue of TMAs we did find only very little CICs in this study and therefore could not investigate senescence involvement in non-professional phagocytosis. Therefore it was not possible to investigate the significance of senescence for CIC in normal tissue. A further limitation is that the cell experiments were only performed with heat and camptothecin treatment. In contrast, the patients were treated with radiochemotherapy. However, it was not our primary aim to perform the same treatment on cells and patients, but to induce senescence and to study senescence as a trigger for non-professional phagocytosis.

## Conclusion

Senescent cells can be non-professionally phagocytized and at the same time they can non-professionally phagocytize in vivo. In vitro experiments indicate that it is unlikely that senescence is a strong trigger for non-professional phagocytosis. Combined high rates of non-professional phagocytosis and high rates of senescence are an extremely poor prognostic factor for overall survival.

## Material and methods

### Cell cultures

Three different cell lines were studied in vitro for senescence and sixteen cell lines for cell-in-cell structures. Cancer cells were pancreatic adenocarcinoma cells BxPC-3, glioblastoma cells U87, U138, U251 and T98G (American Type Culture Collection) and head and neck cancer cells HN-p26. Primary fibroblast cell lines p26F, SBLF-4, − 7, − 9, − 10, − 11, − 12, − 13, − 22, − 23 were each derived from young Caucasians. Human bronchial epithelial cells (BEAS-2B) were obtained from the British Sigma/Public Health Consortium. BxPC-3 cells were cultured in RPMI-1640 medium (Life Technologies GmbH, Darmstadt, Germany) and fibroblast cell lines in F12 medium (Life Technologies GmbH, Darmstadt, Germany). Fetal calf serum, penicillin/streptomycin, glutamine and non-essential amino acids were added in different concentrations for each culture. The cells were cultured in at 37 °C, 5% CO_2_ and 95% humidity.

### Staining and immunofluorescence

Senescence was determined either by flow cytometry or immunostaining as two independent methods. For the flow cytometric analysis, cells were incubated for 30 min in Bafilomycin A1 and afterwards C_12_FDG (Invitrogen, Auckland, New Zealand) was added for 60 min at 37 °C. For senescence analysis by immunostaining anti-H3K9me3 (Millipore, Darmstadt, Germany), anti-HMGA2 and p21 (Cell Signaling, Leiden, Netherlands) antibodies were used. Cytoskeleton was stained by α–tubulin (Abcam, Cambridge, UK). Secondary antibodies were Alexa 488 chicken anti mouse or Alexa 594 chicken anti rabbit (Molecular Probes, Darmstadt, Germany). Phagocytosis experiments were performed using CellTrace Oregon Green (CTOG) (Invitrogen, Auckland New Zealand) for the living cells and CellTrace Far Red (CTFR) (Invitrogen, Auckland, New Zealand) for heat (56 °C) for camptothecin treated cells. Cell nuclei were stained by DAPI (Roche, Grenzach-Wyhlen, Germany) and slides were mounted using Vectashield (Vector Laboratories Inc., Burlingame, USA). Cell images were acquired by a Zeiss Axio Plan 2 fluorescence microscope (Zeiss, Göttingen, Germany). Nuclear size was analyzed with Biomas software (MSAB, Erlangen, Germany).

### Necrosis, senescence induction and non-professional phagocytosis

Senescence was induced in cells by 120 and 200 nM camptothecin for up to 7 days. Necrosis was induced by heating cells in a hyperthermic bath at 56 °C for 45 min. Cell death was analyzed by flow cytometry using the Annexin V-APC and 7AAD staining. The portion of β-galactosidase positive, living and necrotic cells were analyzed by flow cytometry (Gallios Flow Cytometer, Beckman Coulter, Fullerton, USA). Non-professional phagocytosis was studied by coincubating the same number of red and green cells for 4 h. Homotypic phagocytosis experiments were always performed. The living cells and the dead cells in one experiment always came from the same cell line. For the phagocytosis trials the following cell mixtures were used: Vital green cells/heat-treated red cells, vital green cells/senescent red cells, senescent red cells/heat-treated green cells, vital green cells/vital red cells. Slides were scanned with a fluorescence microscope at 400x magnification (Carl Zeiss Microscopy, Göttingen, Germany) and Metafer4 as software (Metasystems, Altlußheim, Germany). At least 1000 vital cells were counted to calculate the cell-in-cell rates. In case of cell-in-cell rates of less than 1% a total of 3000 viable cells were counted.

### Human specimens, tissue micro arrays and immunohistochemical staining

The frequency and prognostic relevance of cell-in-cell structures and senescent cells were studied in a cohort of 192 rectal cancer patients that received neoadjuvant radiochemotherapy (RCT). All samples were processed into tissue microarrays (TMAs) of 2 mm core diameter. Clinical data were obtained from the Erlangen Tumor Centre Database and from patient’s records (Table 1).

The average age was 64 and 63 years for pre-RCT and post-RCT specimens, respectively. The samples named “biopsies” (*n* = 96) were collected 6 weeks prior to RCT. Concurrent chemotherapy most frequently consisted of 5-fluorouracil/oxaliplatin. A post-treatment Dworak tumor regression score of grade 3 was achieved in most cases (*n* = 54.1%). Six weeks after the end of neoadjuvant RCT, patients had surgical tumor resection as part of routine clinical treatment (*n* = 146). The neoadjuvant radiochemotherapy and the time intervals correspond to the standard of treatments. Samples from the surgical specimen were taken from the “central tumor” region, the “invasive front” as well as surrounding “normal tissue”. Immunohistochemical staining were performed on formaldehyde-fixed paraffin embedded tissue microarrays. Tissue sections were dewaxed. The antigens were damasked using target removal solutions pH 6 (TRS6, Dako Cytomation) in a steam cooker. Anti-E-cadherin (BD, Heidelberg, Germany) was used to visualize cell membranes by an alkaline-phosphatase-labeled polymer kit (Zytochem-Plus AP-PolymerKit, Zytomed Systems, Berlin, Germany) and a fast-red color reaction (Sigma-Aldrich, Deisenhofen, Germany). Senescent cells were detected by anti-H3K9me3 antibodies and a fast-blue color reaction (Sigma-Aldrich, Deisenhofen, Germany).

TMA Slides were scanned with a microscope at 200x magnification (Carl Zeiss Microscopy, Göttingen, Germany) using Metafer4 software (Metasystems, Altlußheim, Germany). Intraepithelial and stromal areas of each TMA spot were identified by the image processing software Biomas. The senescent cells and cell-in-cell structures were manually selected. Biomas software recorded the counts in the intraepithelial area per mm^2^. Every cell-in-cell structure was photographed and stored. Cells were regarded as senescent, when their nuclei stained positive for H3K9me3. Cell-in-cell structures were defined as complete enclosure of an inner cell by the membrane of an outer cell, a round nucleus of the inner cell and a semilunar enclosing by the nucleus of the outer cell.

Survival data were censored to 72 months to avoid dilution of data by untraceable drop-out of individual study participants. A corresponding cut-off was determined by a ROC curve analysis. Local failure-free survival, metastasis-free-survival, tumor-specific survival and overall survival were determined using the Kaplan Meier method and the log rank test. Univariate and multivariate Cox regression analysis for overall survival was performed. Age, gender, TNM stage, grading, high cell-in-cell counts/mm^2^, high senescence counts/mm^2^ and the combination of high cell-in-cell and high senescence counts/mm^2^ were evaluated.

### Statistical analysis

IBM SPSS Statistics version 21 (IBM Corp, Armonk, NY, USA) was used for statistical analysis. Data were expressed as mean ± standard deviation (SD). Differences between individual groups were analyzed by Mann-Whitney U, Fisher’s exact test or Student’s t-test. Survival curves for overall survival, tumor specific survival, local recurrence free survival and metastasis free survival were plotted using the Kaplan–Meier method and compared with the log-rank test. Optimal cut-off points for survival analysis between low or high density groups of senescent cells or cell-in-cell events were determined through receiver operating characteristic (ROC) curve analysis. Cox proportional hazards model was used to calculate hazard ratios of cell-in-cell rates, senescence rates and clinicopathological characteristics. Covariates with *p* < 0.35 in univariate analysis were included in multivariate Cox regression. The proportional hazards assumption was tested by visual inspection of log minus log curves and was found to be satisfactory for all multivariate covariates. *p*-values < 0.05 were considered to be statistically significant.

## Supplementary Information

**Additional file 1: Figure 1**. Induction of p21+ cells by CPT in BxPC-3, SBLF-7 and SBLF-4 cell lines. Senescence induction by 120 nM Camptothecin for 5 days in a pancreas carcinoma cell line and two skin fibroblasts cell cultures. (A) Representative images of the stained nuclei (dapi), senescent staining (p21) and combined images (merge). (B) Percentage of p21 positive cells of untreated and Camptothecin treated cells. Differences were analyzed by a two-tailed unpaired Mann-Whitney U.

**Additional file 2: Figure 2**. Prognostic significance of intraepithelial senescent cell density/mm^2^ and cell-in-cell phenomena density/mm^2^ in Kaplan Meier plots for tumor specific survival. The cut-off values were determined by the ROC curve analysis. This resulted in specific cut off values for each individual analysis. Five-year survival rates are given in brackets after the designation of the corresponding cut-off values. (A) Senescent cell density in tissue micro arrays of biopsies, (B) central tumor (C) invasive tumor front and (D) normal tissue from tumor resection. (E) Cell-in-cell density in tissue micro arrays of biopsies, (F) central tumor (G) invasive tumor front and (H) normal tissue from tumor resection. Statistical significance was checked by the log-rank test.

**Additional file 3: Figure 3**.Prognostic significance of intraepithelial senescent cell density/mm^2^ and cell-in-cell phenomena density/mm^2^ in Kaplan Meier plots for local recurrence free survival. The cut-off values were determined by the ROC curve analysis. This resulted in specific cut off values for each individual analysis. Five-year survival rates are given in brackets after the designation of the corresponding cut-off values. (A) Senescent cell density in tissue micro arrays of biopsies, (B) central tumor (C) invasive tumor front and (D) normal tissue from tumor resection. (E) Cell-in-cell density in tissue micro arrays of biopsies, (F) central tumor (G) invasive tumor front and (H) normal tissue from tumor resection. Statistical significance was checked by the log-rank test.

**Additional file 4: Figure 4**. Prognostic significance of intraepithelial senescent cell density/mm^2^ and cell-in-cell phenomena density/mm^2^ in Kaplan Meier plots for metastasis free survival. The cut-off values were determined by the ROC curve analysis. This resulted in specific cut off values for each individual analysis. Five-year survival rates are given in brackets after the designation of the corresponding cut-off values. (A) Senescent cell density in tissue micro arrays of biopsies, (B) central tumor (C) invasive tumor front and (D) normal tissue from tumor resection. (E) Cell-in-cell density in tissue micro arrays of biopsies, (F) central tumor (G) invasive tumor front and (H) normal tissue from tumor resection. Statistical significance was checked by the log-rank test.

## Data Availability

The datasets used and/or analyzed during the current study are available from the corresponding author on reasonable request.

## Abbreviations

SASP: Senescence-associated secretory phenotype
CIC: Cell-in-cell
BEAS2B: Epithelial lung cells
SBLF-4 and SBLF-7: Fibroblast cell lines
CPT: Camptothecin
CTFR: CellTrace Far Red
CTOG: CellTrace Oregon Green
DAPI: 4′,6-Diamidin-2-phenylindol
7AAD: 7-Aminoactinomycin
β-gal: β-galactosidase
HMGA2: High-mobility group AT-hook 2 protein
H3K9me3: Histone 3 tri-methylated at the 9th lysine residue
RCT: Radiochemotherapy
TMA: Tissue Micro Array
TRS6: Target removal solutions pH 6
ROC: Receiver operating characteristic analysis
