# Supplementary material for: Role of tumor cell senescence in non-professional phagocytosis and cell-in-cell structure formation
Source: BMC Mol Cell Biol. 2020 Nov 7;21:79. doi: 10.1186/s12860-020-00326-6 (PMC7648987; doi:10.1186/s12860-020-00326-6)
Supplement: Supplementary file 1 — Additional file 1: Figure 1. Induction of p21+ cells by CPT in BxPC-3, SBLF-7 and SBLF-4 cell lines. Senescence induction by 120 nM Camptothecin for 5 days in a pancreas carcinoma cell line and two skin fibroblasts cell cultures. (A) Representative images of the stained nuclei (dapi), senescent staining (p21) and combined images (merge). (B) Percentage of p21 positive cells of untreated and Camptothecin treated cells. Differences were analyzed by a two-tailed unpaired Mann-Whitney U. [file 12860_2020_326_MOESM1_ESM.pdf]

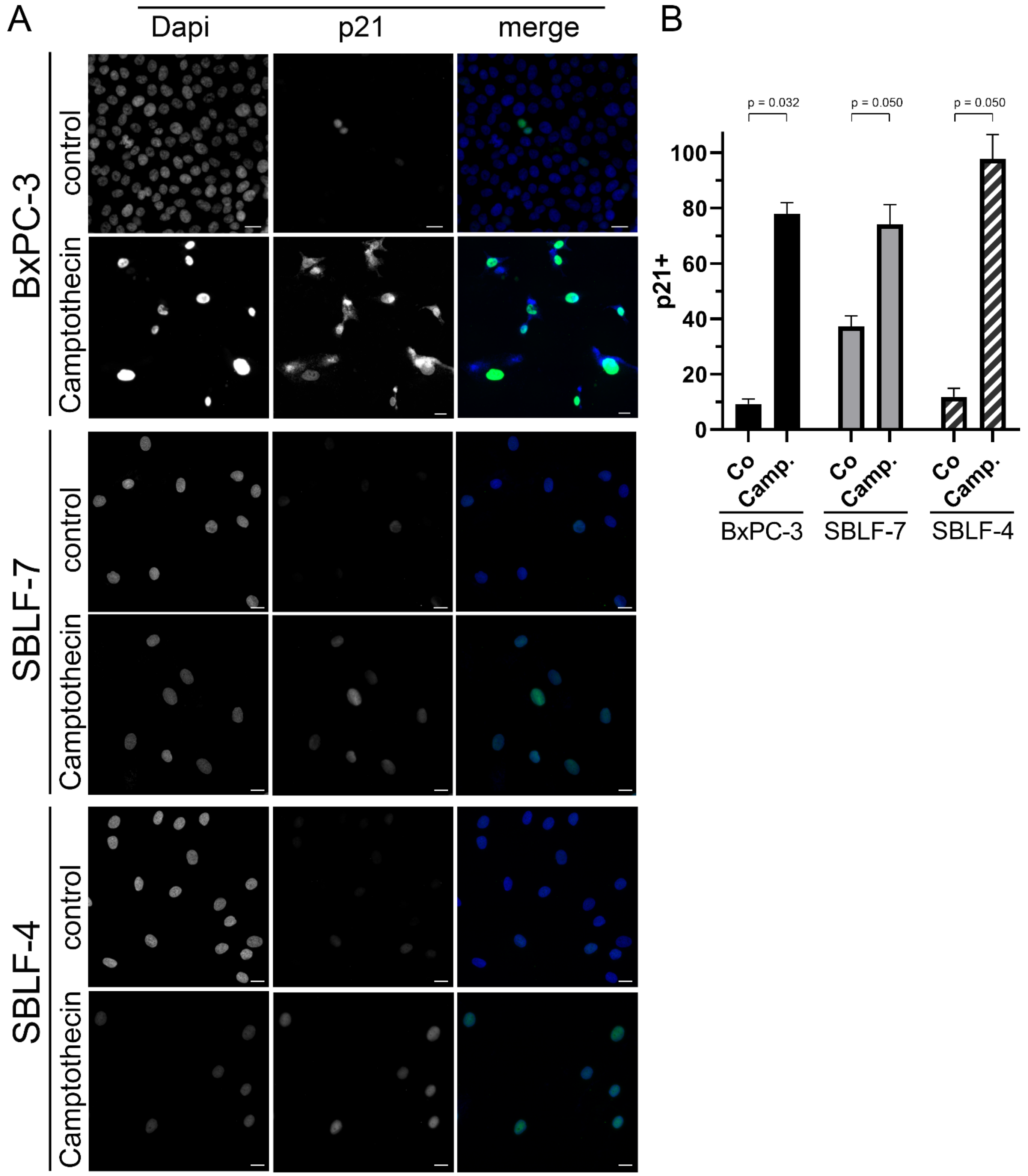

Additional figure 1: Induction of p21+ cells by CPT in BxPC-3, SBLF-7 and SBLF-4 cell lines

Senescence induction by 120 nM Camptothecin for 5 days in a pancreas carcinoma cell line and two skin fibroblasts cell cultures. (A) Representative images of the stained nuclei (dapi), senescent staining (p21) and combined images (merge). (B) Percentage of p21 positive cells of untreated and Camptothecin treated cells. Differences were analyzed by a two-tailed unpaired Mann-Whitney U.
