# Supplementary material for: Role of tumor cell senescence in non-professional phagocytosis and cell-in-cell structure formation
Source: BMC Mol Cell Biol. 2020 Nov 7;21:79. doi: 10.1186/s12860-020-00326-6 (PMC7648987; doi:10.1186/s12860-020-00326-6)
Supplement: Supplementary file 4 — Additional file 4: Figure 4. Prognostic significance of intraepithelial senescent cell density/mm2 and cell-in-cell phenomena density/mm2 in Kaplan Meier plots for metastasis free survival. The cut-off values were determined by the ROC curve analysis. This resulted in specific cut off values for each individual analysis. Five-year survival rates are given in brackets after the designation of the corresponding cut-off values. (A) Senescent cell density in tissue micro arrays of biopsies, (B) central tumor (C) invasive tumor front and (D) normal tissue from tumor resection. (E) Cell-in-cell density in tissue micro arrays of biopsies, (F) central tumor (G) invasive tumor front and (H) normal tissue from tumor resection. Statistical significance was checked by the log-rank test. [file 12860_2020_326_MOESM4_ESM.pdf]

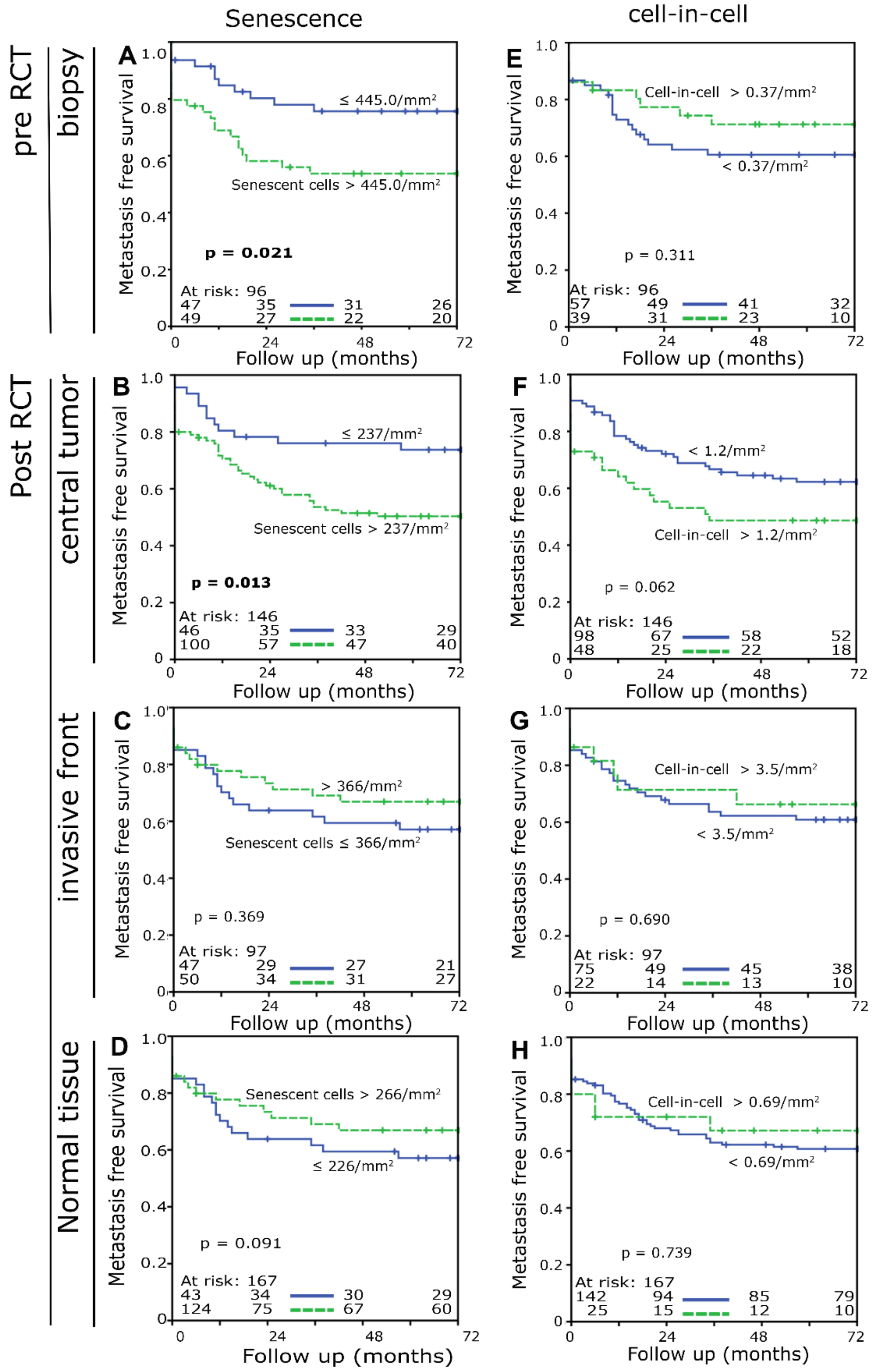

Additional figure 4: Prognostic significance of intraepithelial senescent cell density/mm² and cell-in-cell phenomena density/mm² in Kaplan Meier plots for metastasis free survival.

The cut-off values were determined by the ROC curve analysis. This resulted in specific cut off values for each individual analysis. Five-year survival rates are given in brackets after the designation of the corresponding cut-off values. (A) Senescent cell density in tissue micro arrays of biopsies, (B) central tumor (C) invasive tumor front and (D) normal tissue from tumor resection. (E) Cell-in-cell density in tissue micro arrays of biopsies, (F) central tumor (G) invasive tumor front and (H) normal tissue from tumor resection. Statistical significance was checked by the log-rank test.
